# Korean and Chinese citizens’ pandemic fatigue and related factors amidst the prolonged COVID-19 pandemic: Implications for risk communication

**DOI:** 10.1371/journal.pone.0329262

**Published:** 2025-08-13

**Authors:** Yubin Lee, Chenyuan Qin, Minjung Lee, Jie Deng, Jue Liu, Myoungsoon You

**Affiliations:** 1 Department of Public Health, Graduate School of Public Health, Seoul National University, Seoul, Republic of Korea; 2 Department of Epidemiology and Biostatistics, School of Public Health, Peking University, Beijing, China; 3 Dental Research Institute, School of Dentistry, Seoul National University, Seoul, Republic of Korea; 4 Institute for Global Health and Development, Peking University, Beijing, China; 5 National Health Commission Key Laboratory of Reproductive Health, Peking University, Beijing, China; 6 Institute of Health and Environment, Seoul National University, Seoul, Republic of Korea; Universiti Sains Malaysia - Kampus Kesihatan, MALAYSIA

## Abstract

Pandemic fatigue has emerged as a significant public health challenge, particularly in countries that implemented prolonged COVID-19 public health and social measures (PHSM). Understanding the factors contributing to pandemic fatigue and its impact on adherence to health protective behaviors is essential for sustaining public engagement in long-term disease management. This study examines pandemic fatigue in China and South Korea, two countries that maintained prolonged COVID-19 public health and social measures, to identify key predictors and explore its relationship with health protective behaviors. Online surveys were conducted in March 2023 to measure pandemic fatigue levels. To examine the relationships between pandemic fatigue, its predictors (perceived risk, efficacy beliefs, and daily life changes), and health protective behaviors, linear regression and mediation effect analyses were performed. The results indicated that the level of pandemic fatigue was 3.67 in South Korea and 3.47 in China, which was higher than previous research. High efficacy beliefs were associated with lower pandemic fatigue in both countries, while daily life changes had mixed effects. Pandemic fatigue has a significant impact on the adoption of health protective behaviors, with the exception of the practice of resting when unwell. The findings highlight the necessity of reinforcing efficacy beliefs through risk communication for a sustainable pandemic response. Given that risk perception declined over time, traditional fear-based health messages may be less effective in prolonged pandemics. Instead, risk communication strategies that emphasize a sense of control and provide clear, actionable guidance may help sustain public engagement. Furthermore, addressing the daily life changes faced by citizens and creating environments that facilitate the adoption of health-protective behaviors (e.g., access to paid sick leave) are important as long-term pandemic strategies.

## 1. Introduction

In May 2023, the World Health Organization (WHO) officially lifted the Public Health Emergency of International Concern (PHEIC) for COVID-19, marking a significant transition in the global pandemic response [1]. The WHO advised countries to transition from acute responses to long-term and sustainable strategies for managing the disease, incorporating lessons learned from the pandemic [2]. The sustainable approach includes continual adjustment of public health and social measures (PHSM) to reduce SARS-CoV-2 transmission without causing major daily disruptions. A cornerstone of this approach is the continued adherence to basic PHSM, such as hand hygiene and respiratory etiquette, even when SARS-COV-2 transmission or severity is low or non-existent [3].

However, maintaining compliance with basic PHSM has been challenging due to pandemic fatigue, a subjective state characterized by a lack of motivation to engage in recommended health protective behaviors or to seek COVID-19-related information, in the context of prolonged pandemic [4,5]. This phenomenon arises as individuals become accustomed to the pandemic and perceive the threat of infection as lower, while the costs of pandemic response (e.g., economic loss and daily life inconvenience due to restrictions) exceed the perceived risk of infection [4]. Previous studies have identified various factors associated with pandemic fatigue, such as severity of epidemic, levels of policy stringency, and pandemic-related worries and risk perception [4–6].

China and Korea were the two countries affected by the initial outbreak of the COVID-19 and responded successfully with stringent PHSM in the early stages of the pandemic [7]. The Chinese Government maintained a strict ‘zero-COVID’ policy for over two years, which kept infection numbers low but imposed heavy economic and psychological burdens [8]. The dynamic zero-COVID policy were relaxed swiftly in November 2022 and eliminated by early December [8]. South Korea launched the “Gradual Return to Normal Plan” in November 2021, which was suspended due to the Omicron spike [9]. After the number of confirmed patients decreased, the government gradually lifted COVID-19 mandated PHSM [10]. Both countries’ authoritative strategies for COVID-19 responses and prolonged mandatory PHSM, compared to Western countries, likely contributed to pandemic fatigue [11].

Although pandemic fatigue is an essential public health issue to manage, empirical research on the phenomenon is limited, particularly after the emergence of the Omicron surge. Specifically, few studies have examined how pandemic fatigue evolved in prolonged pandemic conditions and how risk communication strategies can be adapted to mitigate its effects. This study investigates pandemic fatigue in two Asian countries, China and Korea, examining how it developed in each country. This research explores the key determinants of pandemic fatigue and their impact on adherence to health protective behaviors. The findings offer insights into risk communication strategies to sustain public engagement in public health and social measures amid the prolonged pandemic.

## 2. Materials and methods

### 2.1. Study design and participants

An online cross-sectional survey was administered from March 15 to March 30, 2023, three years after the WHO designated COVID-19 a pandemic officially [12]. This was the period when the SARS-CoV-2 Omicron variant had spread and become the dominant variant. China ended its zero-COVID strategy in December 2022, which had been in place for nearly three years. This decision was made in light of the declining severity of the COVID-19 pandemic and the mounting socioeconomic and political costs of restrictive PHSM. The mandatory PHSM relaxation resulted in the explosion of SARS-CoV-2 infections, which also affected Korea [8]. By the survey period, weekly confirmed cases were under 70,000 in both countries, and most COVID-19 mandatory PHSM had shifted from mandatory to advisory [13]. The two countries’ COVID-19 public health and social measures are shown in supplementary material (S1 Table in S1 File).

The surveys were administered through the online platforms *Wen Juan Xing* and *Hankook Research*, both of which are well-established research firms maintaining large and diverse respondent pools in China and South Korea. Stratified quota sampling was employed to ensure the representation of key demographic characteristics in both countries. Quotas were established for geographic regions in both countries, with age and gender additionally incorporated in South Korea. This approach aimed to approximate the population distribution in each country and reduce potential sampling bias.

The sample sizes were proportionally adjusted based on the population sizes of two countries. The sample size for this study was determined based on previous studies on risk perception and preventive behaviors during the COVID-19 outbreak in China and South Korea [14,15], using PASS software 15.0 (NCSS LLC., Kaysville, U.T., USA). The calculation was performed considering standard deviations (SDs) of 0.68 in China and 0.58 in Korea, a two-tailed 95% confidence interval, and aiming for a small-to-moderate effect size. This yielded minimum required sample sizes of 711 for China and 517 for Korea. The sample sizes were proportionally adjusted based on the population sizes of the two countries, ensuring a demographically representative sample from each nation. After implementing quality control measures and manual inspection, the final dataset consisted of 4,000 valid responses (3,000 from China and 1,000 from Korea), which exceeds these minimum thresholds and ensures a robust sample for analysis.

Participants were eligible if they were 18 years or older and capable of completing the online questionnaire in their native language. Potential respondents were invited through email and social media advertisements, and the respondents accessed the survey through a Uniform Resource Locator (URL) link. As the survey was conducted online, electronic informed consent was obtained before participation. Participants were explicitly informed about the study’s purpose, procedures, confidentiality protections, and their right to withdraw at any time. The consent process was conducted through an online form, where individuals were required to click the “agree” button to proceed with the survey; those who clicked “disagree” were unable to continue. To protect participant privacy, no personal identifiable information was collected, and all respondents consented to allow their anonymized data to be used for academic purposes. This study was conducted in accordance with the Declaration of Helsinki and was approved by the Institutional Review Board of Seoul National University (IRB No. 2302/002–003). Due to ethical and privacy considerations approved by the Institutional Review Board (IRB), the dataset cannot be made publicly available. However, access may be granted upon reasonable requests. For detailed information on the data access request process, please refer to the dedicated Data Availability section.

### 2.2. Measures

The predictors included three variables: perceived risk, efficacy beliefs, and daily life changes during the COVID-19 pandemic. Perceived risk of disease infection comprised of perceived susceptibility and severity [16]. Participants rated their likelihood of infection and severity if infected on a 5-point Likert scale (1 = “very unlikely (not at all serious)” to 5 = “very likely (very serious)”). Efficacy beliefs comprised two dimensions: self-efficacy and response efficacy [17]. Participants rated their perceived ability to practice protective behaviors and the perceived effectiveness of these behaviors on a 5-point Likert scale (1 = “Strongly disagree” to 5 = “Strongly Agree”). Daily life changes were assessed using a single item, asking participants to indicate the degree of daily life disruption from 0 to 100 compared to their previous daily life [18]. In China, participants rated changes on a scale from 0 (“not changed or even better”) to 100 (“completely worse than before”), whereas in Korea, an 11-point scale was used ranging from 0 (“not changed”) to 100 (“completely changed or disrupted”). Despite this difference in response formats, a higher score indicated a greater degree of disruption or deterioration. A sensitivity analysis was conducted to address potential concerns arising from this operational difference.

Pandemic fatigue was measured using the Pandemic Fatigue Scale (PFS), which is a valid measurement that consists of six items with two subfactors: information fatigue and behavioral fatigue [5]. The items include: “I am tired of all the COVID-19 discussions in TV shows, newspapers, and radio programs, etc.,” and “I feel strained from following all of the behavioral regulations and recommendations around COVID-19.” Responses were rated on a 7-point Likert scale (1 = “Strongly disagree” to 7 = “Strongly agree”), with higher scores indicating greater pandemic fatigue. The internal consistency of PFS in this research was good (Cronbach’s alpha = 0.892 for China and 0.894 for Korea).

Health protective behaviors were measured using five self-reported items: wearing facial masks indoors; wearing facial masks outdoors; hand hygiene; staying home when unwell; and avoiding crowded places. Participants indicated the frequency with which they engaged in health protective behaviors during the previous week (1 = “never”, 2 = “sometimes”, 3 = “often”, and 4 = “always”).

### 2.3. Statistical analyses

Descriptive analyses were performed to investigate participant characteristics and main variables, presented as the frequency, mean (*M*), and standard deviation (*SD*). Multivariate analyses were conducted to identify the relation between pandemic fatigue, its predictors, and health protective behaviors. First, multiple linear regression analysis was performed to identify the factors that are related to pandemic fatigue. Relations between pandemic fatigue and health protective behaviors were examined, controlling for sociodemographic and health-related factors. Additionally, mediation effects of pandemic fatigue between predictors and health protective behaviors were calculated using the process R package (v. 0.2.8). The significance of the direct and indirect path coefficients was estimated using a bootstrapping method; the coefficient was considered significant if the 95% CI did not span zero. The daily life change variable was scaled down by dividing by 10 for the analysis. To assess the potential impact of the different response formats for the daily life change measure, we conducted a sensitivity analysis. For this analysis, the responses from Chinese participants were rounded to the nearest 10-point increment to align with the format used in Korea. All analyses in this study were performed using R v. 4.3.2 (R Foundation for Statistical Computing).

## 3. Results

### 3.1. Characteristics of participants in China and South Korea

A total of 4,000 participants were enrolled in the study. The participants’ sociodemographic and health-related characteristics are presented in Table 1.

**Table 1:** Sociodemographic and health-related characteristics of participants.

	China		South Korea	
	N (3000)	% (100.0)	N (1000)	% (100.0)
Gender				
Male	1225	40.8	495	49.5
Female	1775	59.2	505	50.5
Age (Mean (SD))	31.0 (7.76)		47.8 (15.2)	
18 ~ 29	1385	46.2	166	16.6
30 ~ 39	1271	42.4	149	14.9
40 ~ 49	249	8.3	183	18.3
50 ~ 59	81	2.7	195	19.5
≥ 60	14	.5	307	30.7
Education †				
High school diploma	552	18.4	573	57.3
Bachelor’s degree and above	2448	81.6	427	42.7
Marital status				
Unmarried, Divorced or Widowed	797	26.6	433	43.3
Married	2203	73.4	567	56.7
Household size				
Solitary	227	7.6	160	16.0
2	299	10.0	287	28.7
3	1317	43.9	263	26.3
≥4	1157	38.6	290	29.0
Residence				
Urban	1957	65.2	853	85.3
Rural	1043	34.8	147	14.7
Household income ‡				
1	27	.9	177	17.7
2	166	5.5	356	35.6
3	548	18.3	230	23.0
4	1197	39.9	120	12.0
5	1062	35.4	117	11.7
Subjective Health Status				
Bad	113	3.8	142	14.2
Moderate	997	33.2	529	52.9
Good	1890	63.0	329	32.9
COVID-19 Infection				
None	310	10.3	386	38.6
More than once	2690	89.7	614	61.4

† Junior College in China was classified as part of the “high school diploma” group in this study.‡ Household income level was classified into five levels: China (after-tax monthly household income per capita): 0-999, 1000-2999, 3000-4999, 5000-9999, and ≥ 10,000 yuan.South Korea (pre-tax monthly household income): 0-2 million, 2-4 million, 4-6 million, 6-8 million, and ≥ 8 million won.

In China, the mean age of participants was 31.0 years, and 40.8% identified as men. The majority (81.6%) had a college degree or higher. Among the Chinese respondents, more than 73.4% were married and 82.5% lived in household with three or more members. A total of 65.2% resided in urban areas. Regarding health status, 63.0% of Chinese participants rated their health as “good.”, and more than 8 out of 10 people (89.8%) had infected the COVID-19.

In South Korea, the participants’ mean age was 47.8 years, and 59.5% of them were male. Less than half (47.7%) achieved an education at the college level or beyond. More than half of the Korean respondents were married (56.7%) and lived with three or more household members (55.3%). A total of 85.3% resided in urban areas. Among the Korean participants, less than half (32.9%) reported their health as “good. The COVID-19 infection rate among the Korean participants was 61.4%.

### 3.2. Pandemic fatigue, predictors, and health protective behaviors

**Fig 1:**
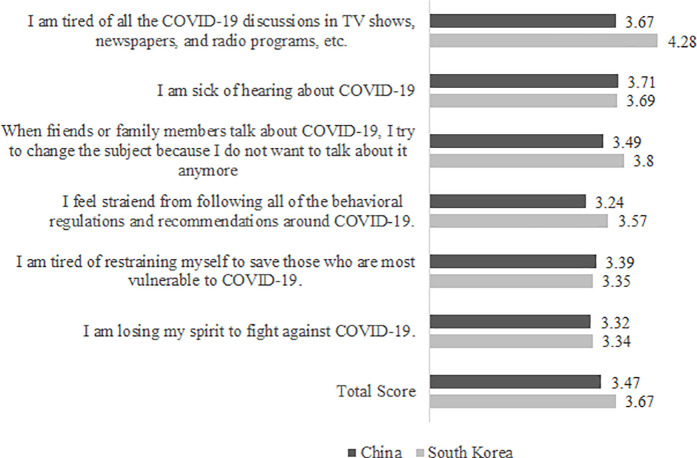
Responses to Pandemic Fatigue Scale (PFS). Mean scores for each item and total mean score on the pandemic fatigue scale (PFS) in China and South Korea.

The levels of pandemic fatigue of Chinese and Korean participants are shown in Fig 1. The overall mean score on the pandemic fatigue was 3.47 for China and 3.67 for Korea. In both countries, the most prominent indicators of pandemic fatigue included “being sick of hearing about COVID-19” (*M*_China_ = 3.71, *M*_Korea_ = 3.69), “being tired of all the COVID-19 discussions in media” (*M*_China_ = 3.67, *M*_Korea_ = 4.28), and “not wanting to talk about COVID-19 anymore and changing the topic” (*M*_China_ = 3.49. *M*_Korea_ = 3.80).

Table 2 presents the descriptive analyses on factors influencing pandemic fatigue and adherence to health protective behaviors. Participants in the study did not perceive themselves as highly susceptible to the coronavirus, nor did they perceive the severity of disease infection as high; the mean score for perceived risk in the two countries was approximately 3 of 5 on a Likert scale. In China, perceived susceptibility had a mean score of 3.39, while perceived severity was lower at 2.90. In Korea, the mean score for perceived susceptibility was 2.84, whereas perceived severity was slightly higher at 3.11.

**Table 2:** Descriptive analysis of main study variables.

	China (N = 3,000)		South Korea (N = 1,000)	
	Mean	SD	Mean	SD
Pandemic Fatigue Predictors				
Perceived susceptibility	3.39	1.01	2.84	0.95
Perceived severity	2.90	0.89	3.11	0.90
Self-efficacy	4.41	0.73	4.29	0.76
Response efficacy	4.42	0.73	4.20	0.81
Daily life change	28.7	19.6	43.2	21.0
Health protective behaviors				
Wearing masks outdoor	3.45	0.75	3.37	0.93
Wearing masks indoor	2.06	0.85	3.13	0.97
Hand hygiene	3.18	0.78	3.26	0.86
Staying home if unwell	2.73	0.87	3.09	0.93
Avoiding crowded places	3.01	0.87	2.85	0.96

Both Chinese and Korean participants reported strong efficacy beliefs, with mean scores exceeding 4.0. In China, the mean scores for self-efficacy and response efficacy were 4.41 and 4.42, respectively. In Korea, the corresponding values were 4.29 and 4.20. With respect to daily life changes, the Korean participants’ responses (*M* = 43.2) were near the median (somewhat changed), while the Chinese participants’ responses (*M* = 28.7) were close to “no change or even better (0 points)”. Among the health protective behaviors, “wearing masks outdoors” (*M*_China_ = 3.45, *M*_Korea_ = 3.37) was the most frequently practiced behavior in both countries, followed by “hand hygiene” (*M*_China_ = 3.18, *M*_Korea_ = 3.26).

### 3.3. Relation between pandemic fatigue, predictors, and health protective behaviors

#### 3.3.1. Factors associated with pandemic fatigue.

The results of multiple linear regression analysis on pandemic fatigue are shown in Table 3. In China, being female (*β* = −0.060), having a large family (*β* = −0.04), having a high household income (*β* = −0.040), and having good subjective health status (*β* = −0.07) were associated with lower levels of pandemic fatigue. In Korea, being older (*β* = −0.11) and having good subjective health status (*β* = −0.07) were associated with lower pandemic fatigue levels. In addition, self-efficacy (*β*_China_ = −0.20, *β*_Korea_ = −0.09) and response efficacy (*β*_China_ = −0.16, *β*_Korea_ = −0.20) were negatively associated with pandemic fatigue in both countries. The level of daily life changes was significantly associated with pandemic fatigue in both countries, but in opposite directions (*β*_China_ = 0.07, *β*_Korea_ = −0.07). In China, individuals who experienced fewer disruptions in daily life were less likely to report pandemic fatigue, whereas in Korea, those with fewer disruptions were more likely to experience it. The sensitivity analysis, which employed the recoded Chinese data, yielded highly consistent results, suggesting that the differences in the response formats did not substantially affect the associations (S2 Table in S1 File).

**Table 3:** Results of multivariate linear regression analysis on pandemic fatigue.

	China			South Korea		
Predictors	*B (SE)*	*β*	*p-value*	*B (SE)*	*β*	*p-value*
Gender ^a^	−0.16 (0.05)	−0.06	**<0.001** ^***^	−0.05 (0.08)	−0.02	0.50
Age	−0.00 (0.00)	−0.03	0.22	−0.01 (0.00)	−0.11	**0.01** ^**^
Education ^b^	−0.05 (0.06)	−0.02	0.42	−0.06 (0.08)	−0.02	0.46
Marital status ^c^	−0.08 (0.07)	−0.03	0.20	0.18 (0.10)	0.08	0.06
Household size	−0.07 (0.03)	−0.04	**0.02** ^*^	−0.04 (0.04)	−0.03	0.35
Residence ^d^	0.04 (0.05)	0.02	0.40	0.09 (0.10)	0.03	0.39
Income	−0.06 (0.03)	−0.04	**0.03** ^*^	−0.06 (0.03)	−0.06	0.10
Subjective Health Status	−0.12 (0.03)	−0.07	**<0.001** ^***^	−0.11 (0.05)	−0.07	**0.03** ^*^
COVID-19 Infection ^e^	0.05 (0.08)	0.01	0.56	0.10 (0.08)	0.04	0.21
Perceived susceptibility	0.04 (0.03)	0.03	0.10	−0.06 (0.04)	−0.05	0.15
Perceived severity	0.05 (0.03)	0.04	0.06	0.01 (0.05)	0.01	0.84
Self-efficacy	−0.37 (0.03)	−0.20	**<0.001** ^***^	−0.14 (0.06)	−0.09	**0.03** ^*^
Response efficacy	−0.29 (0.03)	−0.16	**<0.001** ^***^	−0.30 (0.06)	−0.20	**<0.001** ^***^
Daily life change	0.05 (0.01)	0.07	**<0.001** ^***^	−0.04 (0.02)	−0.07	**0.02** ^*^
*R*^2^/ *R*^2^ Adjusted	0.14/ 0.13			0.11/ 0.09		

*Note*.

* *p-value* < 0.05,

** *p-value* < 0.01,

*** *p-value* < 0.001. Reference group:

a. male;

b. high school diploma;

c. unmarried, divorced, or widowed;

d. rural;

e. none.

#### 3.3.2. Pandemic fatigue’s effect on health protective behaviors.

The results of linear regression analyses that examine pandemic fatigue’s effects on health protective behaviors are presented in S3 Table in S1 File. Overall, greater pandemic fatigue was associated with lower adherence to health protective behaviors (*p* < 0.001). In China, pandemic fatigue significantly reduced the likelihood of wearing masks outdoors (*β*_China_ = −0.19), washing hands (*β*_China_ = −0.12), and avoiding crowded places (*β*_China_ = −0.14). In Korea, it significantly associated with wearing masks outdoors (*β*_Korea_ = −0.14) and indoors (*β*_Korea_ = −0.12). However, pandemic fatigue had no effect on the likelihood of staying home if unwell in either country.

Pandemic fatigue also acted as a mediator between key predictors and health protective behaviors. The mediation analysis, detailed in S4a and S4b Table in S1 File, revealed that perceived risk and efficacy beliefs were statistically associated with certain health protective behaviors directly or indirectly through pandemic fatigue (p < 0.001). The sensitivity analysis confirmed that these mediation effects remained robust despite the differences in the response formats for the daily life change measure (S5 Table in S1 File).

## 4. Discussion

Pandemic fatigue has emerged as a critical public health challenge, particularly in countries that implemented prolonged COVID-19 PHSM. Previous research suggests that exposure to prolonged and restrictive pandemic interventions can diminish public motivation to comply with pandemic guidelines, ultimately weaking the effectiveness of public health responses [4,6]. This study examined pandemic fatigue and its impact on health protective behaviors in two Asian countries, China and South Korea, both of which implemented prolonged COVID-19 PHSM. By analyzing the determinants of pandemic fatigue and mediated effects of pandemic fatigue, the current study provides insights into risk communication strategies for sustaining public engagement in long-term public health crises.

This study revealed that pandemic fatigue was present in both China and South Korea, particularly in relation to COVID-19 discussions and media exposure. The overall mean score of pandemic fatigue was 3.47 in China and 3.67 in Korea, which is higher than pandemic fatigue levels reported in Denmark during 2020–2021 [5]. This discrepancy may be due to the prolonged nature of the pandemic and stricter PHSM in China and Korea [5,6,19].

The findings indicate that some predictors of pandemic fatigue were common across both countries. In both China and South Korea, efficacy beliefs were negatively associated with pandemic fatigue, suggesting that individuals who believe in their ability to engage in health protective behaviors and in the effectiveness of those behaviors were less likely to experience pandemic fatigue. Furthermore, self-efficacy and response efficacy also exhibited statistically significant indirect effects on health protective behaviors via pandemic fatigue. These effects were specifically associated with practices such as wearing masks outdoors and maintaining hand hygiene. These results aligns with previous research in Germany [20], which found that perceived efficacy motivated pandemic protective behaviors.

The findings can be interpreted through risk communication models, such as the extended parallel process model (EPPM) and the Risk Perception Attitude (RPA) framework. The EPPM, a predominant message design theory, suggests that fear appeal messages are effective only when individuals perceive a highly severe threat and vulnerability to a threat [21]. The RPA framework, derived from the EEPM model, also points out efficacy beliefs play a stronger role in behavior change among individuals with low risk perception [17]. Given that risk perception was not high (approximately 3 out of 5 points) while efficacy beliefs were high (higher than 4 out of 5 points) in both China and Korea, our findings suggest that fear-appeal messages may not be effective in encouraging health protective behaviors during prolonged pandemics, when perceived risk declines over time. Instead, risk communication strategies that emphasize a sense of control over risk factors and provide specific instructions can be more effective [22,23].

The impact of daily life changes on pandemic fatigue showed opposite trends in the two countries. In China, participants who reported substantial daily life changes due to COVID-19 were more likely to experience pandemic fatigue. The pandemic restrictions caused mental health and well-being to deteriorate and resulted in devastating and uneven effects on populations; vulnerable populations (e.g., those with low income or chronic disease, and micro-business workers) faced greater adverse effects [24]. If health authorities fail to recognize and address the hardship, risk communication efforts that urge people to adopt the recommended behaviors will be ineffective and people may lose motivation [4]. Therefore, effective risk communication should identify and address these barriers, particularly for vulnerable groups [4]. Engaging various vulnerable groups or their representatives (e.g., civil society organizations) in decision-making processes may help develop more feasible strategies for sustaining public adherence to health guidelines [25,26].

In contrast, in South Korea, lower levels of daily life disruption were associated with greater pandemic fatigue. The Korean government began easing COVID-19 restrictions in November, and by the time of this study, most mandated PHSM had been lifted, except in medical institutions and pharmacies. It is possible that those in professions with persistent exposure to COVID-19, such as healthcare workers, may have felt a stronger sense of obligation to follow health protective behaviors and stay informed about the pandemic, mitigating their fatigue levels.

Another key finding was that pandemic fatigue was significantly associated with reduced adherence to health protective behaviors, though the specific behaviors affected varied by countries. In China, individuals experiencing higher pandemic fatigue were less likely to wear masks outdoors, wash their hands frequently, and avoid crowded places. In Korea, pandemic fatigue primarily affected mask-wearing behaviors, both indoors and outdoors. Notably, pandemic fatigue had no significant effect on the likelihood of staying at home when feeling unwell in either country. During the pandemic, mandatory quarantines were implemented for confirmed patients; however, some workers – particularly those lacking access to paid sick leave or being self-employed – could not afford to take time off when sick [27,28]. For sustainable pandemic responses, governments should address sickness presenteeism by establishing a statutory paid sick leave system and raising awareness of its importance [27,28].

Although this study provides valuable insights, some limitations should be acknowledged. First, its cross-sectional design precludes causal inferences, meaning our regression and mediation findings reflect statistical associations rather than temporal or causal pathways. This design also inherently limits our ability to capture the dynamic nature of pandemic fatigue and its evolving relationships, suggesting a need for future longitudinal studies to establish causal mechanisms more robustly. Second, a minor measurement inconsistency in daily life changes between countries existed. While sensitivity analysis indicated that this inconsistency did not substantially affect the main findings, results related to this variable should be interpreted cautiously. Third, our reliance on self-reported data via an online survey format may have introduced response and sampling biases. Although we employed stratified quota sampling and validated instruments to mitigate these issues, a notable demographic imbalance, particularly concerning age, remained. This potentially undermines the generalizability of our findings, highlighting the importance of future studies incorporating statistical adjustments like post-stratification weighting. Lastly, this study focused on individual-level factors, while broader structural and cultural determinants (e.g., pandemic severity, policy stringency, and cultural norms) also likely influence pandemic fatigue. Future research is encouraged to investigate pandemic fatigue within diverse cultural contexts and to examine how these cultural, political, and structural factors jointly influence its development.

Despite these limitations, this study provides significant insights into pandemic fatigue among Chinese and Korean citizens and offers practical suggestions for improving risk communication during public health emergencies.

## 5. Conclusions

As countries transition into a long-term and sustainable disease management phase, sustaining basic public health and social measures (PHSM) remains a critical challenge. This study enhances our understanding of pandemic fatigue by identifying its key determinants and examining its influence on health protective behaviors in China and Korea − two countries with prolonged COVID-19 PHSM.

Our research highlights that self-efficacy and response efficacy were identified as critical factors related to pandemic fatigue, suggesting that risk communication messages that foster a sense of control over risk factors may be effective in sustaining engagement with basic PHSM during prolonged pandemics. Additionally, pandemic fatigue negatively affected the practice of health protective behaviors overall, except for staying home when unwell. This exception may reflect structural challenges, such as limited access to paid sick leave, which can prevent full adherence of PHSM. Addressing such barriers is essential for fostering sustainable compliance with health guidelines.

In conclusion, this study underscores the importance of addressing both individual and social factors in managing pandemic fatigue for future pandemic responses and preparedness. Risk communication strategies should focus on strengthening efficacy beliefs and acknowledging the daily hardships faced by citizens. Furthermore, supportive environments (e.g., improved labor policies) are essential for ensuring that individuals can adopt and maintain health-protective behaviors during prolonged public health crises.

## Supporting information

S1 File **S1 Table**. COVID-19 PHSMs in two countries as of March 2023. **S2 Table**. Multivariate linear regression on pandemic fatigue in China: sensitivity analysis for daily life changes measurement. **S3 Table.** Results of linear regression examining the effect of pandemic fatigue on health protective behaviors. **S4 Table**. **a** Direct and indirect effects of predictors on health protective behaviors via pandemic fatigue (China). **b** Direct and indirect effects of predictors on health protective behaviors via pandemic fatigue (Korea). **S5 Table.** Mediation analysis of pandemic fatigue in China: sensitivity analysis for daily life changes measurement.
(DOCX)
