# Supplementary material for: Korean and Chinese citizens’ pandemic fatigue and related factors amidst the prolonged COVID-19 pandemic: Implications for risk communication
Source: PLoS One. 2025 Aug 13;20(8):e0329262. doi: 10.1371/journal.pone.0329262 (PMC12348996; doi:10.1371/journal.pone.0329262)
Supplement: S1 File — S1 Table. COVID-19 PHSMs in two countries as of March 2023. S2 Table. Multivariate linear regression on pandemic fatigue in China: sensitivity analysis for daily life changes measurement. S3 Table. Results of linear regression examining the effect of pandemic fatigue on health protective behaviors. S4 Table. a Direct and indirect effects of predictors on health protective behaviors via pandemic fatigue (China). b Direct and indirect effects of predictors on health protective behaviors via pandemic fatigue (Korea). S5 Table. Mediation analysis of pandemic fatigue in China: sensitivity analysis for daily life changes measurement. (DOCX) [file pone.0329262.s001.docx]

**Supplementary Materials**

S1 Table. COVID-19 PHSM in two countries as of March 2023

|  | **China** | **South Korea** |
| --- | --- | --- |
| Outdoor Mask mandate | Wearing masks outdoors was not mandated. | Lifted outdoor mask mandate (Sep. 26, 2022) |
| Indoor mask mandate | The policies differed depending upon the region or location. For example, wearing a mask in public transportation was recommended in some cities, including Guangzhou and Chengdu, but was mandated in other cities, including Beijing.  In schools, teachers and students were not mandated to wear masks, but staff such as medical, security, courier, etc. were mandated when on duty (Mar. 13, 2023). | Lifted indoor mask mandate, except in medical institutions, pharmacies, and public transportation (Jan. 30, 2023)  Lifted mask mandate for public transportation, but still mandated for some medical institutions and pharmacies (Mar. 20, 2023) |
| Quarantine mandate | Lifted quarantine mandate for confirmed patients (Jan. 8, 2023) | 7-day mandatory quarantine period for confirmed patients |
| Social (Physical) distancing mandate | Lifted lockdowns | Lifted all social distancing mandate (Apr. 18, 2022) |
| COVID-19 subsidies | Optimizing financial subsidies for the cost of COVID-19 treatments, implemented up to March 31, 2023 (Jan. 6, 2023).  Increase funding for epidemic prevention and control, focusing on subsidies for patient treatment costs, temporary work subsidies for healthcare workers, vaccinations, as well as expenditures for improving medical treatment capacity (Jan. 13, 2023). | COVID-19 emergency living subsidies for confirmed patients and paid sick leave subsidies for companies were reduced on July 11, 2022, but still provided support to low-income groups and small businesses. |

**S2 Table. Multivariate linear regression on pandemic fatigue in China: sensitivity analysis for daily life changes measurement**

| China | | | |
| --- | --- | --- | --- |
| Predictors | *B (SE)* | *β* | *p-value* |
| Gender ^a^ | -0.16 (0.05) | -0.06 | **<0.001***** |
| Age | -0.00 (0.00) | -0.03 | 0.22 |
| Education ^b^ | -0.05 (0.06) | -0.02 | 0.42 |
| Marital status ^c^ | -0.08 (0.07) | -0.03 | 0.20 |
| Household size | -0.07 (0.03) | -0.04 | **0.02*** |
| Residence ^d^ | 0.04 (0.05) | 0.02 | 0.40 |
| Income | -0.06 (0.03) | -0.04 | **0.03*** |
| Subjective Health Status | -0.12 (0.03) | -0.07 | **<0.001***** |
| COVID-19 Infection ^e^ | 0.05 (0.08) | 0.01 | 0.56 |
| Perceived susceptibility | 0.04 (0.03) | 0.03 | 0.10 |
| Perceived severity | 0.05 (0.03) | 0.04 | 0.06 |
| Self-efficacy | -0.37 (0.03) | -0.20 | **<0.001***** |
| Response efficacy | -0.29 (0.03) | -0.16 | **<0.001***** |
| Daily life change | 0.05 (0.01) | 0.07 | **<0.001***** |
| *R*^2^ / *R*^2^ Adjusted | 0.14 / 0.13 | | |

**S3 Table. Results of linear regression examining the effect of pandemic fatigue on health protective behaviors**

|  | China | | | | South Korea | | | |
| --- | --- | --- | --- | --- | --- | --- | --- | --- |
| Outcomes | B (SE) | β | *p*-value | R^2^ | B (SE) | β | *p*-value | R^2^ |
| Wearing masks outdoor | -0.12 (0.01) | -0.19 | <0.001*** | 0.05 | -0.11 (0.02) | -0.14 | <0.001*** | 0.10 |
| Wearing masks indoor | -0.02 (0.01) | -0.03 | 0.06 | 0.02 | -0.09 (0.03) | -0.12 | <0.001*** | 0.05 |
| Hand hygiene | -0.07 (0.01) | -0.12 | <0.001*** | 0.09 | -0.04 (0.02) | -0.05 | 0.08 | 0.07 |
| Staying home if unwell | -0.01 (0.01) | -0.02 | 0.39 | 0.02 | -0.04 (0.03) | -0.05 | 0.11 | 0.03 |
| Avoiding crowded places | -0.09 (0.01) | -0.14 | <0.001*** | 0.03 | -0.04 (0.03) | -0.05 | 0.14 | 0.05 |
| *Note*. **p-value* < 0.05, ***p-value* < 0.01, ****p-value* < 0.001  Control variables: gender, age, education, marital status, household size, residence, income, subjective health status, COVID-19 infection | | | | | | | | |

**S4a Table.** **Direct and indirect effects of predictors on health protective behaviors via pandemic fatigue (China)**

| Predictors → Pandemic Fatigue → Health protective Behavior | | **Wearing masks outdoor** | **Wearing masks indoor** | **Hand hygiene** | **Staying home if unwell** | **Avoiding crowded places** |
| --- | --- | --- | --- | --- | --- | --- |
| **Perceived susceptibility** | Total Effect  (95% Bootstrap CI) | 0.028  (-0.003 – 0.057) | 0.028  (-0.006 – 0.058) | 0.011  (-0.017 – 0.043) | 0.005  (-0.031 – 0.038) | -0.001  (-0.035 – 0.035) |
|  | Direct Effect  (95% Bootstrap CI) | **0.033**  **(0.033 – 0.063)** | 0.029  (-0.005 – 0.060) | 0.015  (-0.014 – 0.045) | 0.005  (-0.031 – 0.038) | 0.003  (-0.030 – 0.038) |
|  | Indirect Effect  (95% Bootstrap CI) | -0.005  (-0001 – 0.001) | -0.001  (-0.003 – 0.000) | -0.003  (-0.008 – 0.001) | 0.000  (-0.002 – 0.001) | -0.004  (-0.010 – 0.001) |
| **Perceived severity** | Total Effect  (95% Bootstrap CI) | **0.065**  **(0.033 – 0.096)** | **0.068**  **(0.033 – 0.106)** | 0.029  (-0.001 – 0.060) | **0.064**  **(0.027 – 0.101)** | **0.052**  **(0.014 – 0.089)** |
|  | Direct Effect  (95% Bootstrap CI) | **0.075**  **(0.040 – 0.105)** | **0.071**  **(0.035 – 0.108)** | **0.035**  **(0.005 – 0.066)** | **0.066**  **(0.028 – 0.102)** | **0.060**  **(0.022 – 0.096)** |
|  | Indirect Effect  (95% Bootstrap CI) | **-0.009**  **(-0.016 – -0.003)** | -0.002  (-0.005 – -0.000) | **-0.006**  **(-0.011 - -0.002)** | -0.001  (-0.004 – 0.001) | **-0.008**  **(-0.014 – -0.002)** |
| **Self-efficacy** | Total Effect  (95% Bootstrap CI) | **0.260**  **(0.217 – 0.297)** | 0.028  (-0.016 – 0.068) | **0.222**  **(0.181 – 0.263)** | **0.050**  **(0.007 – 0.092)** | **0.171**  **(0.126 – 0.219)** |
|  | Direct Effect  (95% Bootstrap CI) | **0.224**  **(0.179 – 0.263)** | 0.019  (-0.029 – 0.060) | **0.201**  **(0.158 – 0.244)** | **0.048**  **(0.003 – 0.094)** | **0.137**  **(0.090 – 0.186)** |
|  | Indirect Effect  (95% Bootstrap CI) | **0.036**  **(0.026 – 0.050)** | 0.009  (-0.002 – 0.022) | **0.020**  **(0.010 – 0.032)** | 0.002  (-0.010 – 0.013) | **0.034**  **(0.021 – 0.047)** |
| **Response efficacy** | Total Effect  (95% Bootstrap CI) | **0.171**  **(0.135 – 0.204)** | 0.001  (-0.042 – 0.045) | **0.162**  **(0.117 – 0.202)** | **0.071**  **(0.027 – 0.112)** | **0.132**  **(0.086 – 0.181)** |
|  | Direct Effect  (95% Bootstrap CI) | **0.132**  **(0.094 – 0.169)** | -0.008  (-0.053 – 0.038) | **0.139**  **(0.096 – 0.181)** | **0.071**  **(0.028 – 0.113)** | **0.099**  **(0.052 – 0.149)** |
|  | Indirect Effect  (95% Bootstrap CI) | **0.039**  **(0.028 – 0.050)** | 0.010  (-0.001 – 0.021) | **0.023**  **(0.013 – 0.034)** | 0.000  (-0.010 – 0.011) | **0.033**  **(0.021 – 0.045)** |
| **Daily life change** | Total Effect  (95% Bootstrap CI) | -0.005  (-0.021 – 0.009) | 0.008  (-0.008 – 0.026) | **-0.019**  **(-0.036 - -0.005)** | 0.011  (-0.007 – 0.028) | 0.014  (-0.004 – 0.032) |
|  | Direct Effect  (95% Bootstrap CI) | 0.004  (-0.011 – 0.003) | 0.010  (-0.006 – 0.028) | -0.013  (-0.029 – 0.002) | 0.012  (-0.006 – 0.029) | **0.022**  **(0.005 – 0.040)** |
|  | Indirect Effect  (95% Bootstrap CI) | **-0.009**  **(-0.013 – -0.006)** | -0.002  (-0.004 – 0.000) | **-0.006**  **(-0.009 - -0.004)** | -0.001  (-0.003 – 0.001) | **-0.008**  **(-0.012 – -0.005)** |

**S4b Table. Direct and indirect effects of predictors on health protective behaviors via pandemic fatigue (Korea)**

| Predictors → Pandemic Fatigue → Health protective Behavior | | **Wearing masks outdoor** | **Wearing masks indoor** | **Hand hygiene** | **Staying home if unwell** | **Avoiding crowded places** |
| --- | --- | --- | --- | --- | --- | --- |
| **Perceived susceptibility** | Total Effect  (95% Bootstrap CI) | **0.147**  **(0.086 – 0.204)** | **0.125**  **(0.060 – 0.184)** | **0.079**  **(0.019 – 0.139)** | 0.011  (-0.055 **–** 0.074) | **0.111**  **(0.041 – 0.176)** |
|  | Direct Effect  (95% Bootstrap CI) | **0.138**  **(0.078 – 0.194)** | **0.117**  **(0.050 – 0.179)** | **0.076**  **(0.016 – 0.135)** | 0.007  (-0.058 **–** 0.072) | **0.108**  **(0.040 – 0.174)** |
|  | Indirect Effect  (95% Bootstrap CI) | **0.009**  **(0.001 – 0.020)** | **0.008**  **(0.001 – 0.018)** | 0.003  (-0.001 – 0.009) | 0.004  (-0.001 – 0.010) | 0.003  (-0.002 – 0.010) |
| **Perceived severity** | Total Effect  (95% Bootstrap CI) | **0.176**  **(0.108 – 0.242)** | **0.162**  **(0.090 – 0.233)** | **0.114**  **(0.054 – 0.176)** | 0.035  (-0.034 – 0.102) | **0.143**  **(0.071 – 0.211)** |
|  | Direct Effect  (95% Bootstrap CI) | **0.167**  **(0.099 – 0.233)** | **0.155**  **(0.081 – 0.226)** | **0.111**  **(0.050 – 0.174)** | 0.031  (-0.035 – 0.100) | **0.140**  **(0.069 – 0.208)** |
|  | Indirect Effect  (95% Bootstrap CI) | 0.009  (-0.001 – 0.020) | 0.007  (-0.001 – 0.018) | 0.003  (-0.001 – 0.009) | 0.003  (-0.001 – 0.011) | 0.003  (-0.002 – 0.010) |
| **Self-efficacy** | Total Effect  (95% Bootstrap CI) | **0.299**  **(0.220 – 0.379)** | **0.232**  **(0.149 – 0.313)** | **0.371**  **(0.301 – 0.439)** | **0.183**  **(0.105 – 0.262)** | **0.167**  **(0.082 – 0.249)** |
|  | Direct Effect  (95% Bootstrap CI) | **0.274**  **(0.188 – 0.355)** | **0.209**  **(0.124 – 0.294)** | **0.376**  **(0.303 – 0.446)** | **0.178**  **(0.099 – 0.260)** | **0.161**  **(0.072 – 0.245)** |
|  | Indirect Effect  (95% Bootstrap CI) | **0.025**  **(0.008 – 0.045)** | **0.023**  **(0.005 – 0.045)** | -0.004  (-0.019 – 0.012) | 0.005  (-0.011 – 0.024) | 0.005  (-0.014 – 0.026) |
| **Response efficacy** | Total Effect  (95% Bootstrap CI) | **0.320**  **(0.247 – 0.395)** | **0.264**  **(0.184 – 0.344)** | **0.306**  **(0.243 – 0.373)** | **0.180**  **(0.105 – 0.254)** | **0.202**  **(0.122 – 0.281)** |
|  | Direct Effect  (95% Bootstrap CI) | **0.298**  **(0.224 – 0.374)** | **0.244**  **(0.166 – 0.325)** | **0.311**  **(0.244 – 0.382)** | **0.176**  **(0.101 – 0.253)** | **0.201**  **(0.116 – 0.285)** |
|  | Indirect Effect  (95% Bootstrap CI) | **0.022**  **(0.004 – 0.042)** | **0.020**  **(0.000 – 0.042)** | -0.006  (-0.022 – 0.010) | 0.004  (-0.016 – 0.023) | 0.001  (-0.019 – 0.023) |
| **Daily life change** | Total Effect  (95% Bootstrap CI) | 0.007  (-0.022 – 0.034) | 0.018  (-1.906 – 1.913) | 0.005  (-0.019 – 0.031) | 0.011  (-0.017 – 0.037) | **0.029**  **(0.001 – 0.058)** |
|  | Direct Effect  (95% Bootstrap CI) | 0.002  (-0.027 – 0.028) | 0.014  (-0.015 – 0.044) | 0.003  (-0.021 – 0.029) | 0.009  (-0.020 – 0.036) | 0.027  (-0.000 – 0.056) |
|  | Indirect Effect  (95% Bootstrap CI) | **0.005**  **(0.001 – 0.010)** | **0.004**  **(0.001 – 0.009)** | 0.002  (-0.000 – 0.005) | 0.002  (-0.000 – 0.005) | 0.002  (-0.001 – 0.006) |

**S5 Table. Mediation analysis of pandemic fatigue in China: sensitivity analysis for daily life changes measurement**

| Predictors → Pandemic Fatigue → Health protective Behavior | | **Wearing masks outdoor** | **Wearing masks indoor** | **Hand hygiene** | **Staying home if unwell** | **Avoiding crowded places** |
| --- | --- | --- | --- | --- | --- | --- |
| **Daily life change** | Total Effect  (95% Bootstrap CI) | -0.005 (-0.020 – 0.009) | 0.010  (-0.007 – 0.026) | **-0.019 (-0.035** – **-0.004)** | 0.010 (-0.009 – 0.027) | 0.014 (-4.690 – 3.267) |
|  | Direct Effect  (95% Bootstrap CI) | 0.004 (-0.010 – 0.018) | 0.012 (-0.005 – 0.028) | -0.013 (-0.029 – 0.002) | 0.011 (-0.007 – 0.027) | **0.022 (0.004 – 0.039)** |
|  | Indirect Effect  (95% Bootstrap CI) | **-0.009 (-0.013** – **-0.006)** | -0.002 (-0.004 – -0.000) | -**0.006 (-0.009 – -0.004)** | -0.001 (-0.009 – 0.001) | **-0.008 (-0.012 – -0.005)** |
